# Rare Genetic Diseases with Defects in DNA Repair: Opportunities and Challenges in Orphan Drug Development for Targeted Cancer Therapy

**DOI:** 10.3390/cancers10090298

**Published:** 2018-09-01

**Authors:** Sonali Bhattacharjee, Saikat Nandi

**Affiliations:** Cold Spring Harbor Laboratory, Cold Spring Harbor, New York, NY 11724, USA

**Keywords:** rare disease, orphan drugs, synthetic lethality, targeted cancer therapy, combination therapy, DNA repair, precision medicine, genomic instability, chemotherapy, clinical trials

## Abstract

A better understanding of mechanistic insights into genes and enzymes implicated in rare diseases provide a unique opportunity for orphan drug development. Advances made in identification of synthetic lethal relationships between rare disorder genes with oncogenes and tumor suppressor genes have brought in new anticancer therapeutic opportunities. Additionally, the rapid development of small molecule inhibitors against enzymes that participate in DNA damage response and repair has been a successful strategy for targeted cancer therapeutics. Here, we discuss the recent advances in our understanding of how many rare disease genes participate in promoting genome stability. We also summarize the latest developments in exploiting rare diseases to uncover new biological mechanisms and identify new synthetic lethal interactions for anticancer drug discovery that are in various stages of preclinical and clinical studies.

## 1. Introduction

**Figure 1:**
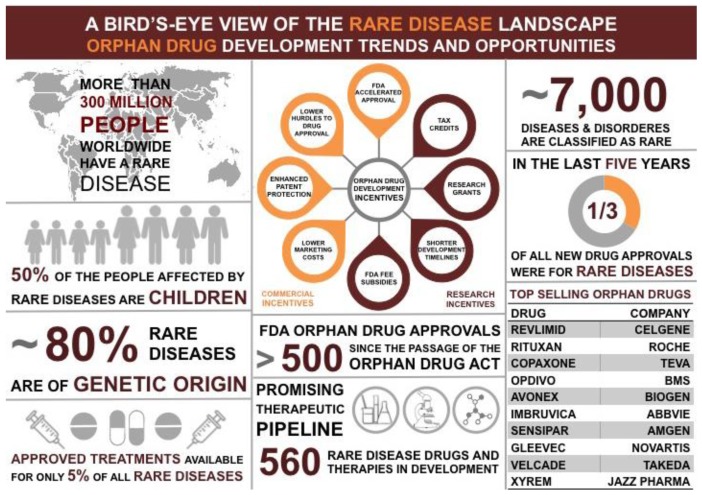
Opportunities and Challenges in developing orphan drugs to prevent, diagnose and treat rare diseases.

Rare diseases are defined as disorders that affect fewer than 200,000 people in the United States [1]. Although these disorders can often be chronic, debilitating, and life-limiting illnesses there are few effective therapies for these diseases (Figure 1) [1]. This is largely because pharmaceutical industries are unable to recover the cost associated with the R&D and marketing of such drugs [1]. The FDA Office of Orphan Products Development (OOPD) focuses on the development of drugs and products for rare diseases (Figure 1). Interestingly, many rare diseases are caused by the altered function of DNA damage response (DDR) genes causing genome instability—a hallmark of cancer. Since DDR genes are crucial for maintaining genome stability, mutations in these genes may have downstream cancer burden associated with them. Hence, understanding the biology of rare DNA repair diseases can help identify novel cancer therapies.

The DDR network includes several DNA repair pathways, which depending on the type of DNA lesion is employed by the cell to orchestrate repair [2,3]. There are several DNA lesions that can affect a cell: (A) single-stranded breaks, (B) base modifications (e.g., methylation, oxidation), (C) base mismatch (e.g., insertions, deletions, translocations), (D) incorporation of bulky adducts during replication (covalent attachment of aryl groups to the DNA), (E) incorporation of abasic sites (where a base is missing from the DNA), (F) interstrand and intrastrand covalent crosslinks, and (G) double-stranded breaks. Due to space limitations, in the following section we briefly discuss the repair of single-strand and double-strand DNA breaks.

### 1.1. Repair of Single-Strand DNA Breaks (SSBs)

SSBs, as the name suggests, afflicts one strand of the DNA double helix and is repaired by one of three pathways; base excision repair (BER), nucleotide excision repair (NER) and mismatch repair (MMR) [4]. BER, NER, and MMR are vital cellular pathways essential for the maintenance of genome integrity and cell survival (reviewed in [5,6,7,8]). Briefly, BER has evolved to repair endogenous DNA damage that can lead to modifications in the DNA bases such as alkylation, oxidation and deamination [5]. NER is distinguished from BER due to its versatility of sensing structural distortions in the double helix rather than specific base modifications [9,10]. “Bulky” lesions generated by UV radiation such as cyclobutane pyrimidine dimers (CPDs) and 6,4-photoproducts (6-4PPs) generate structural distortion in the DNA that obstruct progression of transcription and replication machineries that can lead to genome instability if left unrepaired [11]. MMR is involved in postreplication repair (PRR) and corrects DNA mismatches generated during DNA replication and recombination that escapes the proofreading function of DNA polymerases and can become permanent in dividing cells if left unrepaired. MMR thereby prevents both mutagenesis in the short term and oncogenesis in the long term [12,13].

### 1.2. Repair of Double-Strand DNA Breaks (DSBR)

**Figure 2:**
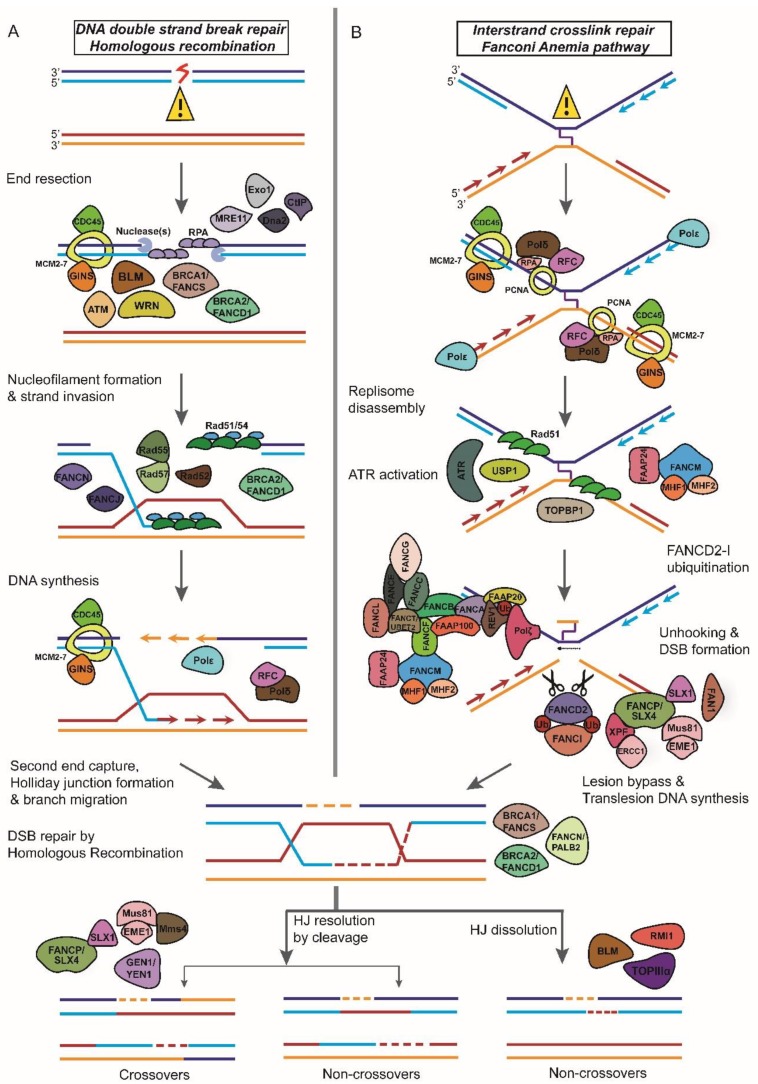
A schematic for DNA damage response and repair Werner, Bloom, ATM, and Fanconi Anemia proteins are members of the DNA damage response network. (**A**) Werner and Bloom helicases and ATM serine/threonine kinase are involved in DNA end processing after the double-strand break is formed. (**B**) Fanconi Anemia proteins are involved in DNA interstrand crosslink sensing and repair by homologous recombination.

The two major DSBR pathways employed by the cell for the repair of broken DNA ends primarily differ in the use of a homologous DNA template for repair. Homologous recombination (HR) is an error-free, template-dependent pathway that uses DNA molecules of “identical or nearly identical nucleotide sequences” as template to copy the information across the double-strand break (DSB) (Figure 2A) [14]. Non-homologous end-joining (NHEJ), does not depend upon sequence homology to recover the lost genetic information at the site of the DNA break and is a mutagenic, error-prone mechanism of DNA repair [11]. A second NHEJ concomitant pathway often referred to as alternative-NHEJ (Alt-NHEJ)/Microhomology-mediated end-joining (MMEJ) differs from NHEJ most significantly in that it uses shorter homology overhangs (5–25 base pair (bp)) on the ends of the double-strand breaks during the alignment of the broken ends before religating them [15]. Though both these pathways are highly conserved throughout evolution, HR appears to be the predominant mechanism of DSBR during embryogenesis (and S/G2 phases of cell cycle), whereas NHEJ is a major pathway for repair in post-natal life and in the G0/G1 phase of the cell cycle [16].

In this review, we highlight how insights from understanding the biology of rare genetic diseases with defects in DNA damage response and repair can be translated to the clinic for cancer therapy. We discuss four diseases—Werner syndrome (WS), Bloom syndrome (BS), Ataxia telangiectasia (AT) and Fanconi Anemia (FA). We highlight the latest research in the development of small molecule inhibitors for these DNA damage repair enzymes that are being used in the clinic either as single agent or in combination with radio- and/or chemotherapy to exploit defects in DNA damage repair pathways in cancer cells. 

## 2. Leveraging Insights from the Biology of Rare Genetic Diseases for Targeted Cancer Therapy 

Geneticist Calvin Bridges first described synthetic lethality as a genetic interaction in which deficiency in a pair of genes results in cell death, while the disruption of either gene is viable [17]. The key impediment to exploiting synthetic lethality in cancer therapy is the identification of robust synthetic lethal genetic interactions [6]. What makes DNA repair genes attractive targets for cancer therapy using the synthetic lethality approach is; (1) many DNA repair genes have synthetic lethal relationships with oncogenes and tumor suppressor genes, (2) many repair genes and repair pathways are upregulated in tumors giving rise to a distinct genetic profile that can be exploited for targeted killing of cancer cells, (3) several cancer cells are dependent on DNA repair pathways for survival in response to genotoxin stress (e.g., radio/chemotherapy) [6,18,19,20,21,22,23,24]. In fact, in recent years, the success of poly(ADP ribose) polymerase inhibitors (PARPi): small molecules that inhibit the molecular and cellular function of PARP-1 and in doing so exploits the synthetic lethal relationship between PARP and BRCA1 or BRCA2 in BRCA1/2-null (ovarian or breast) tumors in response to platinum-based chemotherapy has made chemically induced synthetic lethality more mainstream in the development of new generation anti-cancer drugs [25,26]. 

PARP-1 is a nuclear enzyme that senses and binds to single-stranded DNA breaks and catalyzes the formation of PARP polymers by a process known as poly(ADP-ribosyl)ation (PARylation) [27]. PARP-1 uses the PAR chains to serve as a docking platform for the recruitment of DNA repair factors to the site of the break; in particular, PARP-1 recruits the BER core-complex proteins such as XRCC1, Polβ and DNA ligase III to orchestrate repair [27,28]. PARP-1 inhibition leads to an accumulation of SSBs, which if left unrepaired lead to collapse replication forks that are essentially DSBs and rely on HR for replication restart and repair [29]. *BRCA1* and *BRCA2* are tumor suppressor genes that are also key HR repair protein [29]. Due to their synthetic lethal relationship, in HR-defective BRCA1/2-mutant cells, that is, cancer cells, chemical inhibition of PARP-1 is selectively toxic in combination with conventional chemotherapy [29].

Applying the principle of chemically induced synthetic lethality to identify targets for cancer therapy has immense potential and relies on the identification of inhibitors that can selectively modulate DNA repair proteins that are either directly or indirectly involved in carcinogenesis. In the following section, we discuss four DNA repair genes/gene networks: Werner helicase, Bloom helicase, ATM kinase and Fanconi Anemia proteins and how pharmacologically inhibiting these gene products in combination with a cancer driver mutation is synthetically lethal and can be exploited for developing orphan drugs.

## 3. Werner Syndrome

WS is an adult-onset, autosomal-recessive, representative segmental progeroid syndrome [30]. Dr. Otto Werner at the Royal Albrecht University of Kiel first described WS in 1904 [31]. The clinical phenotypes of WS include an early onset of aging symptoms such as bilateral cataracts, abnormal glucose and lipid metabolism, hypogonadism, atherosclerosis, diabetes, skin ulcers, osteoporosis, and other age-related disorders [32,33]. Individuals with WS show no observable phenotype in their first decade. Generally, the first sign of WS is a lack of the pubertal growth spurt during teenage years [34]. WS is a rare genetic disorder with an estimated global incidence ranging between 1 in 1,000,000 and 1 in 10,000,000 births; however, demographically the incidence is higher in Japan at 1 in 100,000 births [35]. WS is caused by a mutation in the WRN gene that encodes a 180 kDa RecQ type DNA/RNA helicase that possesses both helicase and exonuclease activities (Figure 2A) [36]. WS is associated with abnormalities in many DNA metabolic pathways such as DNA repair, replication, and telomere maintenance [36]. 

### 3.1. Werner Helicase in Non-Homologous End-Joining and Base Excision Repair

WRN plays a regulatory role in pathway choice during DSBR by stimulating NHEJ and inhibiting MMEJ [37]. DNA-PKcs and its regulatory subunit, Ku70/80, initiates NHEJ repair in response to DNA damage that results in the formation of DSBs [38]. The Ku70/80 complex has been shown to physically interact with WRN and enhance its exonuclease activity without affecting its helicase activity [39]. The processing of the broken DNA ends by WRN generates substrates suitable for ligation by XRCC4 and ligaseIV [38].

WRN knockdown cells show sensitivity to DNA damaging agents (mitomycin C, camptothecin, and hydrogen peroxide) and reduced BER activity [40,41,42]. BER proceeds either via short-patch (SP-BER), in which one single nucleotide, or via long-patch (LP-BER), where greater than one nucleotide is incorporated during the repair process [43]. WRN has been shown to physically and functionally interact with proteins involved in both SP-BER and LP-BER. Notably, the strand displacement activity of DNA Polymerase β (Pol β), involved in both SP-BER and LP-BER is stimulated by WRN helicase activity [44,45,46]. WRN helicase activity cooperates with its (1) proofreading activity to assist Pol β during LP-BER and (2) exonuclease activity to process BER intermediates with 3′-mismatches [41,47]. WRN also interacts with other enzymes involved in SP-BER and LP-BER, including apurinic/apyrimidinic endonuclease 1 (APE1), flap endonuclease 1 (FEN-1) and poly(ADP-ribose) polymerase 1 (PARP-1), among others [48,49,50]. 

### 3.2. Small Molecule Inhibitors and Werner Helicase

Modulation of DNA repair proteins by small molecule inhibitors as targets for anticancer therapy has attracted great interest in recent years. The WRN helicase is one of these DNA repair factors. Two small molecule inhibitors from the National Cancer Institute (NCI) Diversity Set, NSC 19630 and NSC 617145 have been identified as pharmacological inhibitors of WRN helicase activity [51,52]. NSC 19630 [1-(propox-ymethyl)-maleimide] has been reported to inhibit growth of cell lines in various cancers with a notably strong representation in leukemia and renal cancer [52]. NSC 617145 [1,1′-(2,2-Dimethyl-1,3-propanediyl)bis[3,4-dichloro-1H-pyrrole-2,5-dione] is reported to be a candidate for chemically inducing synthetic lethality in Fanconi Anemia-deficient tumors harboring autosomal recessive mutations in either the FA group A (FANCA) or group D (FANCD2) genes [51]. FA-A and FA-D2 cells show hypersensitivity to low concentrations of MMC when co-treated with NSC 617145. This is because NSC 617145 acts synergistically with MMC and exacerbates MMC-induced DNA damage that accumulates in the absence of a functionally intact FA pathway. NSC 19630 and NSC 617145 are both attractive drugs for developing improved anticancer treatment regimens.

## 4. Bloom Syndrome

BS is an autosomal recessive chromosomal instability syndrome [53]. The clinical phenotypes of BS include prenatal and postnatal growth retardation, sunlight sensitivity, immune deficiency, fertility defects and a predisposition to the development of cancer, most commonly leukemia and lymphoma [54,55]. BS is a rare genetic disorder with fewer than 300 cases reported. However, epidemiologically, one-third of people with the disease are of Eastern European Jewish (Ashkenazi) decent and a disease prevalence of approximately 1:48,000 [53,56,57]. BS is due to mutations of the *BLM* gene (15q26.1), which encodes the BLM DNA helicase. BLM is a member of the conserved RecQ helicase family, an enzyme involved in maintenance of genomic integrity (Figure 2A) [58,59]. Due to its helicase activity, BLM, like WRN, functions to facilitate several metabolic processes that involve unwinding the DNA helix including DNA replication, RNA transcription, and DNA repair [60].

### 4.1. Bloom Helicase and Homologous Recombination

BLM protein displays a DNA-dependent ATPase activity and 3′–5′ DNA helicase activity that can unwind a variety of DNA substrates that arise during HR-mediated DNA repair. Accurate processing of these DNA intermediates is crucial for the maintenance of chromosomal stability and genomic integrity [61]. BLM, along with its conserved partner enzymes, including topoisomerase IIIα, RMI1 and 2 can also branch migrate recombination intermediates like DNA displacement loops (D-loop) and Holliday junctions (HJs) and channel these DNA molecules away from pathways leading to crossover products (Figure 2A) [62,63,64,65,66,67]. In the absence of BLM, HR is still functional albeit unregulated; this results in an increase in the number of sister chromatid exchanges (SCE) which arise by HR between sister chromatids during the S or G2 phases of the cell cycle that are characteristic of the syndrome [59,68,69]. 

### 4.2. Small Molecule Inhibitors and Bloom Helicase

The compound, designated ML216, identified from a high-throughput screen of a chemical compound library and medicinal chemistry optimization, is a potent small molecule inhibitor of BLM [54]. This inhibitor acts through competitive inhibition of the DNA binding activity of BLM [54]. In cell-based assays, using cultured human fibroblasts; ML216 induces SCEs, elevated sensitivity to aphidicolin (an inhibitor of replicative DNA polymerases) and reduced proliferation [54]. However, these phenotypes were not observed in otherwise isogenic, BLM-deficient cells [54]. These observations warrant further studies for the potential development of a new class of chemotherapy drugs to treat tumors that rely on BLM for proliferation.

Cu/Zn superoxide dismutase 1 (SOD1) is a non-essential gene that acts as a regulator of antioxidant defense [70]. Published reports suggest a synthetic lethal relationship between BLM and SOD1 in colorectal cancer (CRC) cell models [71]. Using siRNA-based silencing, chemical inhibition of SOD1 by ammonium tetrathiomolybdate (ATTM) and Lung Cancer Screen-1 (LCS-1), the authors report the induction of selective killing within *BLM*-deficient cells [71]. This data identifies SOD1 as a novel drug target in *BLM* CRC contexts.

## 5. Ataxia Telangiectasia

A-T is a rare, recessive genetic disorder that is primarily an immunodeficiency disease and affects a number of different organs in the body [72]. A-T is characterized by dilated blood vessels (telangiectasia) and a loss of voluntary movement coordination that includes a gait abnormality (ataxia) [72]. A-T is a rare genetic disorder with an estimated global incidence ranging between 1 out of 40,000 and 1 out of 100,000 births worldwide with no reported geographical or demographical epidemiology (data from NCI). A-T is due to mutations of the *ATM* gene, a member of the phosphatidyl inositol 3-kinase-like family of serine/threonine protein kinases (PIKKs) [73,74]. The ATM gene is located on chromosome 11q 22–23, includes 66 exons, and encodes a 370 kDa serine/threonine protein kinase [75]. ATM mediates checkpoint control by inducing cell cycle arrest at G1/S, S and G2/M phases in cells exposed to ionizing radiation (IR) and other agents that produce DSBs. In response to DNA damage, several proteins are phosphorylated at Ser/Thr-Glu motifs and additional sites in an ATM-dependent manner [76,77,78,79]. ATM also exerts cell cycle delay, in part, by phosphorylation and activation of downstream effector kinases, like Checkpoint kinase 2 (Chk2), which, in turn, phosphorylates p53 and several other targets [80,81].

### 5.1. ATM and Double-Strand Break Repair

In response to DSBs, Mre11-Rad50-Nbs1 (MRN) complex is one of the first factors recruited to the site (Figure 2A). Once recruited, MRN recruits ATM to the DNA lesion by binding to the C terminus of NBS1 and subsequently stimulates ATM kinase activity [82,83,84,85]. Following the activation of ATM by broken DNA ends, ATM rapidly phosphorylates S139 in the C-terminal tail of histone variant H2AX to form γH2AX [86]. The protein Mdc1 mediates the recruitment of ATM to the chromatin flanking the DSBs [87]. Through its C-terminal tandem BRCT domains, Mdc1 directly binds to γH2AX and through its FHA domain Mdc1 interacts with ATM [87,88,89,90]. In addition, Mdc1 recruits MRN to chromatin by an interaction with the FHA-BRCT region of NBS1, thereby stimulating the phosphorylation of ATM, which in turn promotes additional γH2AX formation along chromatin [91,92,93]. The phosphorylation of H2AX by ATM around DSBs triggers the chromatin-based signaling cascade involving phosphorylation, ubiquitylation, SUMOylation, and other post-translational modifications to promote recruitment of DNA repair proteins like BRCA1 and 53BP1 to the damage site [94,95,96,97,98,99,100,101,102,103,104,105,106,107]. 

ATM has roles both in NHEJ and HR. NHEJ requires several proteins such as ATM, the MRN complex, DNA-PKcs, and Artemis [108]. ATM is required for the phosphorylation and activation of DNA-PKcs [109]. ATM and DNA-PKcs are also essential for Artemis activation and recruitment to DNA ends [110]. ATM promotes HR by phosphorylating and activating CtIP, a downstream effector molecule important for DSB end resection (Figure 2A). Presence of CtIP at the DNA lesion facilitates the recruitment of replication protein A (RPA) to initiate HR [111,112,113]. Moreover, ATM-dependent phosphorylation of CtIP promotes the removal of Ku from single-ended DSBs and sets in motion Rad51-mediated strand invasion step of HR [114].

### 5.2. Small Molecule Inhibitors and ATM-CHK2 Helicases

Due to their central role in promoting DNA repair and facilitating the resistance of cancer cells to genotoxin treatment, the ATM-CHK2 kinases are excellent candidates for modulation by small molecules inhibitors for targeted cancer therapy. Table 1 summarizes a comprehensive list of small molecule inhibitors of ATM and CHK2 that are in preclinical or clinical development for cancer therapy being tested either as single agents or in combination with radio-and/or chemotherapy.

## 6. Fanconi Anemia

First described by the Swiss pediatrician Guido Fanconi, FA is a rare cancer-susceptibility syndrome caused by biallelic mutations in one of the 21 known complementation groups and is inherited as recessive autosomal or X-linked genetic disease [127,128,129,130,131]. The clinical phenotypes of FA include developmental disorders and physical abnormalities, progressive aplastic anemia, chromosomal fragility, congenital abnormalities and susceptibility to cancer: particularly myeloid leukemia and solid tumors including squamous cell carcinomas of the head and neck, liver tumors, and gynecologic malignancies [129,132,133,134,135]. The incidence rate of FA is estimated to be about 1 in 136,000 live births. This rare condition has a higher incidence among people of Ashkenazi Jewish descent, the Roma population of Spain, and black South Africans due to founder mutations [133]. FA can result from mutations in at least 21 genes. These genes encode for proteins, which along with several associated genes, form a nuclear multiprotein network that orchestrates DNA repair in a common pathway called the FA pathway (Figure 2B) [136,137,138,139,140,141,142,143,144,145,146,147,148,149,150,151].

### 6.1. Fanconi Anemia Proteins and DNA Interstrand Crosslink Repair

DNA interstrand crosslinks (ICLs) are toxic lesions that block replication and transcription by inhibiting the translocation of enzymes on the replication fork by covalently linking strands of the double helix leading to genome instability [136]. There are two major pathways for the repair of ICLs: (1) replication-independent ICL repair and (2) replication-dependent ICL repair [152]. The replication-dependent repair of ICLs is dependent on FA-mediated HR repair (Figure 2B) [152]. 

The FA proteins have been divided into three groups and they act in a sequential manner to promote ICL repair [136]. Group I (also known as the core complex) protein FANCM, a helicase and DNA translocase together with accessory factors Fanconi anemia-associated protein 24 (FAAP24), FAAP 100 and the histone fold proteins MHF1 (FAAP16 or CENPS) and MHF2 (FAAP10 or CENPX) recognizes the ICL and recruits other Group I proteins also known as the core complex consisting of FANCA, FANCB, FANCC, FANCE, FANCF, FANCG, FANCL, FANCT, and FAAP20 to the ICL site (Figure 2B) [153]. Following recruitment to the ICL, two core complex components FANCL (the E3 ligase) and FANCT (ubiquitin E2 ligase) ubiquitinates downstream Group II proteins FANCD2–I (also known as the ID complex) (Figure 2B) [154,155]. The recruitment of the ID complex to the ICL is mediated by UHRF1 (ubiquitin-like with PHD and RING finger domains 1) protein, involved in ICL sensing (Figure 2B) [156]. Ubiquination of the FANCD2–I complex is essential for the recruitment of nucleases to the ICL for promoting nucleolytic incision flanking the crosslink, a phenomenon referred to as ‘unhooking’ of the ICL (Figure 2B) [157]. FANCD2-Ub first recruits the nuclease scaffolding protein SLX4 (FANCP) to the ICL, which then promotes the recruitment of other structure-specific endonucleases like XPF-ERCC1, MUS81-EME1, FAN1, and SLX1 to the site of the ICL (Figure 2B) [158,159,160,161,162]. Following the unhooking step, translesion DNA polymerases such as REV7 (FANCV), and polymerase η inserts a base opposite the unhooked lesion and polymerase ζ extends DNA synthesis from the misincorporated base to fill the ssDNA gaps resulting from ICL unhooking in order to complete replication (Figure 2B) [163,164]. The ssDNA gaps are converted to dsDNA breaks by MUS81–EME1, which is subsequently repaired by HR (Figure 2B) [165].

### 6.2. Small Molecule Inhibitors and Fanconi Anemia Proteins

Chemotherapeutic drugs like cisplatin and its derivatives, carboplatin and oxaliplatin act by inhibiting DNA replication by forming ICLs that impede the progression of the replication fork [166]. The crucial role played by FA proteins in the cellular resistance to ICLs makes them excellent targets for inhibition by small molecule inhibitors that may sensitize cancer cells to radio- and/or chemotherapy [166]. Table 2 summarizes a list of small molecule inhibitors of FANCD2 ubiquitination and foci formation; a marker of FA pathway abrogation that are in preclinical or clinical development for cancer therapy.

## 7. Conclusions

Identifying target genes/enzymes, and understanding the mechanism(s) underlying the progression of rare cancers provides us with the opportunity to: (A) exploit the disease-causing gene as a therapeutic target, and (B) expand the repertoire of targets for orphan drug discovery and development. In recent years, the success of small-molecule inhibitors for cancer therapy over conventional chemotherapy has directed scientific interest in identifying targetable enzymes that can be successfully modulated to improve the efficacy of current drugs. As presented in this review, enzymes implicated in rare genetic disease have been excellent targets for such modulation by small-molecule inhibitors and exploiting vulnerabilities in cancer. It is important to note that this approach also has certain limitations. First, there is a very high failure rate while designing new drugs particularly in late stages of clinical trials and second, many small-molecule inhibitors have high rates of cell toxicity making them unusable in therapy. However, with the advancements in next-generation genome sequencing technologies, identification of reliable biomarkers and large-scale gene profiling methods, the possibility of developing new personalized drugs for cancer patients is on the horizon.

## Figures and Tables

**Table 1:** Small molecule inhibitors of ATM-CHK2 Kinases in preclinical or clinical development for cancer therapy.

Target	Molecule	Stage of Testing	Tumor Type	Reference/ ClinicalTrial.gov Identifier
**CHK1/CHK2**	AZD7762	Phase 1 Administered as single intravenous unit and in combination with gemcitabine	Solid Tumors	NCT00413686
**CHK1/CHK2**	CBP501	Phase 1 Administered in combination with cisplatin/nivolumab	Advanced Solid Tumors	NCT03113188
**CHK1/CHK2**	LY2606368	Phase 1 Administered in combination with cisplatin/cetuximab/pemetrexed/fluorouracil	Neoplasm Metastasis Colorectal Neoplasm Breast Cancer	NCT02124148
**CHK1/CHK2**	LY2606368	Phase 2 Single agent	Ovarian Cancer Breast Cancer Prostate Cancer	NCT02203513
**ATM/ATR**	CGK733	Preclinical testing using Chk1-deficient HBV-positive hepatocellular carcinoma cells	Hepatocellular carcinoma	[115]
**ATM**	CP466722	Preclinical testing using multiple cell lines in combination with infrared radiation (IR)	Cervical cancer Fibroblasts	[116]
**ATM/ATR**	Caffeine	Preclinical testing as single agent using human cancer cells and non-transformed mouse fibroblast cell lines	Breast Cancer Prostate cancer	[117]
**ATM**	KU59403	Preclinical testing using p53 functional and dysfunctional models of human cancer in combination with camptothecin, doxorubicin or etoposide	Osteosarcoma	[118]
**ATM**	KU55933	Preclinical testing using human mesenchymal stem cells in combination with IR	Mesenchymal stem cells	[119]
**ATM**	KU55933	Preclinical testing using ATM-defective and normal human fibroblast cells in combination with doxorubicin	Fibroblast	[120]
**ATM**	KU-60019	Preclinical testing using human glioma cells in combination with IR	Glioma	[121]
**ATM**	KU-60019	Preclinical testing using non-invasive breast cancer cells in combination with doxorubicin	Breast Cancer	[122]
**CHK2**	PV1019	Preclinical testing using human tumor cell lines in combination with topotecan, camptothecin or radiation	Ovarian Carcinoma	[123]
**CHK2**	CCT241533	Preclinical testing using human tumor cell lines in combination with PARP inhibitors	Colon Cancer Breast Cancer Glioblastoma Osteosarcoma Lung Cancer Cervical Cancer	[124]
**CHK1/CHK2**	XL-844	Preclinical testing using HT-29 cell line in combination with IR	Colon Cancer	[125]
**CHK1/CHK2**	XL-844	Preclinical testing using multiple cell lines in combination with gemcitabine	Pancreatic Cancer Cervical Cancer Ovarian Cancer	[126]

**Table 2:** Small molecule inhibitors of FANCD2 mono-ubiquitination in preclinical or clinical development for cancer therapy.

Target	Molecule	Stage of Testing	Tumor Type	Reference/ ClinicalTrial.gov Identifier
**26S proteasome**	Bortezomib	Phase 3 Administered in combination with Daratumumab/bortezomib/dexamethasone	Relapsed or Refractory Multiple Myeloma	NCT03234972
**26S proteasome**	Bortezomib	Phase 3 Administered in combination with daratumumab/cyclophosphamide bortezomib/dexamethasone	Amyloidosis	NCT03201965
**26S proteasome**	Bortezomib	Phase 2 As single agent	Multiple Myeloma	NCT00153920
**26S proteasome**	Bortezomib	Phase 2 As single agent	Multiple Myeloma Stage I, II, and III	NCT00075881
**26S proteasome**	Bortezomib	Phase 2 As single agent	Primary Peritoneal Cavity Cancer and Recurrent Ovarian Epithelial Cancer	NCT00023712
**26S proteasome**	Bortezomib	Phase 2 Administered in combination with bortezomib/vorinostat	Acute Lymphoblastic Leukemia	NCT02553460
**FANCF**	Curcumin	Preclinical testing using cell lines in combination with cisplatin	Ovarian and Breast Cancer	[167]
**FANCS**	Phenylbutyrate	Preclinical testing using cell lines in combination with cisplatin	Head and Neck Cancer	[168]
**USP1**	GW7647	Preclinical testing using cell lines in combination with cisplatin	Non-small-cell Lung Cancer	[169]
**USP1**	Pimozide	Preclinical testing using cell lines in combination with cisplatin	Non-small-cell Lung Cancer	[169]
**USP1-UAF1**	ML323	Preclinical testing using cell lines in combination with cisplatin	Non-small-cell Lung Cancer and Osteosarcoma	[170]
**USP1**	C527	Preclinical testing using cell lines in combination with MMC and camptothecin	Leukemia	[171]
**ATR**	Wortmannin	Preclinical testing using HeLa cell lines in combination with IR or mitomycin C (MMC)	Cervical Cancer	[172]
**PKA** **PKC** **PKG**	H-9	Preclinical testing using HeLa cell lines in combination with IR	Cervical Cancer	[167]
**CDK** **GSK3**	Alsterpaullone	Preclinical testing using HeLa cell lines in combination with IR	Cervical Cancer	[167]

## Abbreviations

Alt-NHEJ	Alternative-NHEJ
ATTM	Ammonium tetrathiomolybdate
AT	Ataxia telangiectasia
BER	Base excision repair
BS	Bloom syndrome
CRC	Colorectal cancer
CPD	Cyclobutane pyrimidine dimers
D-loops	Displacement loops
DDR	DNA damage response
DSBs	Double-strand breaks
DSBR	Double-strand break repair
FA	Fanconi Anemia
OOPD	FDA Office of Orphan Products Development
HJ	Holliday junction
HR	Homologous recombination
ICLs	Interstrand crosslinks
LCS-1	Lung Cancer Screen-1
MMEJ	Microhomology-mediated end-joining
MMR	Mismatch repair
MRN	Mre11-Rad50-Nbs1
NHEJ	Non-homologous end-joining
NER	Nucleotide excision repair
PARP	Poly(ADP ribose) polymerase
PRR	Post-replication repair
RPA	Replication protein A
SSBs	Single-strand breaks
SCE	Sister chromatid exchanges
SOD1	Superoxide dismutase 1
WS	Werner syndrome
